# Osmotic Pump Drug Delivery Systems—A Comprehensive Review

**DOI:** 10.3390/ph15111430

**Published:** 2022-11-18

**Authors:** Yosif Almoshari

**Affiliations:** The Department of Pharmaceutics, College of Pharmacy, Jazan University, Jazan 45142, Saudi Arabia; yalmoshari@jazanu.edu.sa

**Keywords:** osmotic pump, osmosis, controlled release system, novel drug delivery system, osmotic pressure

## Abstract

In the last couple of years, novel drug delivery systems (NDDS) have attracted much attention in the food and pharmaceutical industries. NDDS is a broad term that encompasses many dosage forms, one of which is osmotic pumps. Osmotic pumps are considered to be the most reliable source of controlled drug delivery, both in humans and in animals. These pumps are osmotically controlled and release active agents through osmotic pressure. To a large extent, drug release from such a system is independent of gastric fluids. Based on such unique properties and advantages, osmotic pumps have made their mark on the pharmaceutical industry. This review summarizes the available osmotic devices for implantation and osmotic tablets for oral administration.

## 1. Introduction

Conventional oral drug dosage forms are known to provide a prompt release of active ingredients, but one cannot control the release of the medication and cannot maintain a therapeutic concentration at the desired site for a long time [1]. Various factors affect the rate and extent to which the drug reaches systemic circulation after administration, including the excipients used, physicochemical properties of the active ingredient, physiology and pH of the gastrointestinal tract, gastric emptying rate, and GI motility [2,3]. As the drug concentration in plasma varies with a conventional dosage form, it is challenging to obtain a steady-state drug concentration in plasma. These fluctuating drug levels may hamper the achieving and maintaining of an effective concentration, which may result in an undesirable drug response or may not produce a therapeutic response [4].

To overcome these limitations with conventional dosage forms, controlled-release drug delivery systems, through which the drug is released in a predicted and sustained manner, have been developed. They maintain the drug concentration between the minimum effective concentration (MEC) and maximum therapeutic concentration (MTC) for a prolonged time, ensuring sustained therapeutic action [1,5,6,7,8]. Among the various controlled-release systems, osmotic drug delivery systems provide substantial advantages. Since these systems maintain uniform plasma drug concentrations and provide a prolonged therapeutic response, they minimize the dosing frequency and subsequently improve patient compliance [3]. Moreover, they release drugs at a rate that is independent of physiological factors, including GI motility, food, pH, and the hydrodynamics of the dissolution medium. This results in comparable release patterns and an excellent in vitro/in vivo correlation. Another superior advantage of osmotic systems is that they are suitable for use with drugs that have a broad range of water solubility and are easy and simple to formulate, operate, and scale up [1,9,10,11].

Osmosis can be defined as the net movement of water across a selectively permeable membrane due to pressure [12]. There is a concentration difference of the solute across the membrane, which is semi-permeable, creating pressure. Water is allowed to pass through this membrane, and solute molecules are mostly not allowed to as this membrane is selectively permeable. The pressure applied to the higher-solute-concentration side to resist solvent flow is called the osmotic pressure [13]. Osmosis can also be defined as the movement of water from a region with a higher concentration to a region with a lower concentration across a semi-permeable membrane [14].

Osmotic delivery systems or osmotic pumps are mainly composed of a core containing a drug and an osmogen. These are coated with a semi-permeable membrane containing one or more ports for drug delivery, such that the drug is released over time in the form of a solution or suspension [15]. Oral osmotic systems are composed of a compressed tablet core coated with a semi-permeable membrane through which delivery orifices are created using a laser beam or mechanical drill [10]. These controlled systems are based on osmosis and osmotic pressure and are independent of various gastrointestinal factors. However, it is noteworthy that there are critical factors that influence the design of osmotically controlled drug delivery systems, including the drug solubility, delivery orifices, osmotic pressure, semi-permeable membrane, type and nature of the polymer, membrane thickness, and type and amount of plasticizer [16,17].

### History

Advancements in drug delivery technology from the oral route to a specific target have been made over many decades [18]. Besides the oral route, the vascular route of administration has also been widely used. Drugs administered intravenously benefit from avoiding the acidic and enzymatic gastric environment. However, the drawback of the intravenous route is its invasiveness [19]. The place where the catheter or needle is to be applied also requires proper cleanliness, as there is a risk of infection when the therapy is continued for several weeks [20].

The implanted pump was the next advancement—a device designed for implantation under the skin to bypass the application of the catheter. These devices provide constant drug release [21]. A brief description of its advantages and disadvantages is summarized in Table 1. Further advancements in implantable pumps occurred in the 1970s, at which point they were employed in animals only. Osmosis was the principle of this new approach, and these new pumps could be smaller than other constant-rate pumps [22]. Alza Corporation and Felix Theseus made several alterations and improvements to this concept in the 1970s and 1980s, resulting in the construction of the elementary osmotic pump (EOP) [20].

Osmotic delivery systems were first studied in 1748, and a further achievement came in 1877 when osmotic pressure was measured quantitatively. In 1955, the first implantable osmotic pump was developed by Australian pharmacologists, which was named the Rose–Nelson osmotic pump. In 1973, several modifications were made to this pump by Higuchi and Leeper, who introduced the Higuchi–Leeper osmotic pump [23]. Several patents have been granted for osmotic pump system, suggesting that there is a great demand for these products. Oral osmotic pumps are also called gastrointestinal therapeutic systems, such as the EOP [9]. A patent was granted to Alza Corporation in 1976 for an oral osmotic pump. The osmotic bursting drug delivery system was then developed in 1979 [24].

This system was modified in 1982 by adding a hydrogel layer that has the property of swelling in the presence of fluid. A patent for this was granted [25]. Push–pull osmotic pumps utilized in combination therapy were first introduced in 1984 [26,27]. One year later, in 1985, mechanically drilled orifices were removed, and the resulting product became known as the controlled-porosity osmotic pump system; this was patented the following year [28]. It was then possible to use liquid pharmaceutical agents in osmotic systems, and this type of system was granted a patent in 1995. In these systems, the agents are enclosed in a capsule with a delivery port and a layer of osmogen. A semi-permeable membrane surrounds this [29]. Osmotic-pressure-dependent drug delivery via a capsule with an asymmetric membrane was introduced in 1999 [12]. With all these advancements, a novel formulation was introduced in the form of an ionic-driven pump that depends on the osmotic pressure caused by the ionic concentration, which is proportional to the drug release rate. This has the advantage of allowing us to estimate the drug release rate, which may help in the future when making further improvements to ionic osmotic pumps.

## 2. Basic Components of Osmotic Pump Systems

### 2.1. Drug

Not every administered drug needs to provide a prolonged response, so the osmotic pump system is not suitable for all drugs. Drugs that are indicated for the prolonged treatment of diseases with a biological half-life in the range of 1–6 h are best suited for osmotic systems. Drugs with a biological half-life shorter than 1 h are not good candidates, and, similarly, drugs with a half-life greater than 12 h are also not good candidates for controlled release in an osmotic pump system. The drug’s half-life should be short so that it can be sustained or maintained in plasma, and its prolonged release should be the requirement [38]. To be incorporated into this system, drugs should also be neither highly soluble nor very poorly soluble, and the nature of the drug should be potent for this purpose [39,40].

### 2.2. Osmotic Agent

Osmogens and osmogents are other names for osmotic agents, and they create the osmotic pressure in the osmotic delivery system. When a drug has low solubility, it will be released at a slow, first-order rate; to make this release rate faster, osmotic agents are used in the formulation. These agents generate a high gradient of osmotic pressure within the osmotic system; thus, the rate of drug release increases [41]. The osmotic agents available on the market include lactose, fructose, sorbitol, dextrose, sodium chloride, citric acid, potassium chloride, sucrose, xylitol, and mannitol. Osmogens may also consist of mixtures, such as mannitol + sucrose, dextrose + fructose, sucrose + fructose, dextrose + sucrose, mannitol + fructose, lactose + fructose, mannitol + dextrose, or lactose + dextrose [42]. Drugs with good water solubility can be used as osmotic agents, such as mannitol, glycerol, lactulose, sorbitol, or polyethylene glycol. However, osmogenic salts (e.g., sodium chloride and potassium chloride) and sugars can be incorporated into the formulation if the drug itself does not possess osmogenic activity. Hence, water solubility and osmotic activity are the two most important determinant factors when selecting osmotic agents [43].

### 2.3. Semipermeable Membrane

As the nature of the osmotic system membrane is selectively permeable, the polymer should be selectively permeable, to allow for the passage of water only, and should be impermeable to solutes [44]. In osmotic pump preparation, the polymer that is most commonly and extensively used is cellulose acetate, which is provided in various grades of acetyl content [45]. The grades containing 32% and 38% acetyl content are most commonly employed. The degree of substitution (average no. of hydroxyl groups replaced by substituting groups) determines the acetyl content. Other digestive polymers used for this purpose include cellulose esters, like diacetate, propionate, cellulose acetate, triacetate, and cellulose acetate butyrate. Ethers of cellulose can also be included in this, such as ethyl cellulose [46,47]. The material must have sufficient wet strength to retain the integrity of its dimensions, which is beneficial for the device. The ability of the material to allow water permeation must be sufficient so that the flux rate of water stays within the required range. The transmission rates of water vapor can be calculated to estimate the water flux rates. The biocompatibility of the membrane material should also be considered [48]. Common biocompatible polymers include PEG, HPMA, PGA, chitosan, and dextran. These materials, when used in oral systems, can be ingested and then excreted in feces once the osmotic pump is exhausted. Oral and implant fractions that may have been absorbed are very likely to be eliminated by glomerular filtration in the kidney, provided that they are below the glomerular threshold [49].

### 2.4. Wicking Agent [37]

Wicking agents are substances with the ability to absorb water into the porous network of a delivery system. Their main function is to carry solvent molecules to surfaces inside the core of the osmotic device, thereby creating channels of enhanced surface area. A wicking agent is selected based on its nature, either swellable or non-swellable, and based on its ability to undergo physisorption with solvent molecules. Physisorption is a form of van der Waals interaction where the solvent molecules can loosely adhere to the surface of the wicking agent [50]. Sodium lauryl sulfate, polyvinylpyrrolidone, and colloidal silicon dioxide are examples of such agents [51].

### 2.5. Pore-Forming Agents

A microporous membrane forms due to the presence of pore-forming agents. A pore former can also form walls with micro-sized pores. This pore former leaches out, creating pores as the system operates [52]. Alkaline metal salts such as potassium chloride, sodium chloride, and others may be used as pore-forming agents. Alkaline earth metals such as calcium nitrate and carbohydrates such as fructose and glucose can also be employed for this purpose [53].

### 2.6. Coating Solvents

The solvent system conveys the polymer, which is dispersed or dissolved, and other additives to the substrate surface as its primary function. Solvents that are inert and either organic or inorganic in nature are employed to prepare a polymeric solution [54]. These solvents should not cause adverse actions in the core or other materials. Examples of such solvents include methanol, cyclohexane, methylene chloride, isopropyl alcohol, and water [55].

## 3. Delivery Orifice

The drug is delivered through an orifice via the process of diffusion to achieve an optimal, zero-order drug delivery rate [56]. The cross-sectional area of the orifice must be small, but it should not be too small in order to create hydrostatic pressure in the osmotic system. This means that the orifice must be larger than the minimum size requirement. The optimum size range for a delivery orifice is 600 μm to 1 mm [57,58,59]. The delivery orifice can be created via a simple mechanical drilling method [60]. Another technique used to create orifices in the sub-millimeter range is laser drilling technology. The laser beam generally used for this purpose is CO_2,_ and it has proved to be beneficial and economical [61,62]. Leaching materials can also be employed for creating in situ orifices or pores in the membrane [63]. There is a process known as indentation in core tablets that utilizes updated compression punches. The upper punch contains needles for the creation of orifices [64].

## 4. Types of Osmotic Drug Delivery Systems

**Figure 1:**
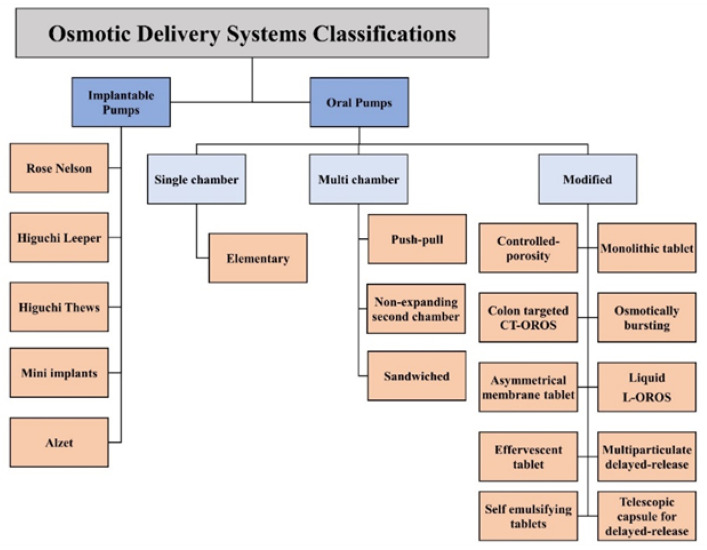
Classification of osmotic delivery systems.

Depending on the active ingredient, design, and purpose of use, osmotic systems are principally classified as shown in Figure 1.

### 4.1. Implantable Systems

#### 4.1.1. The Rose–Nelson Pump

**Figure 2:**
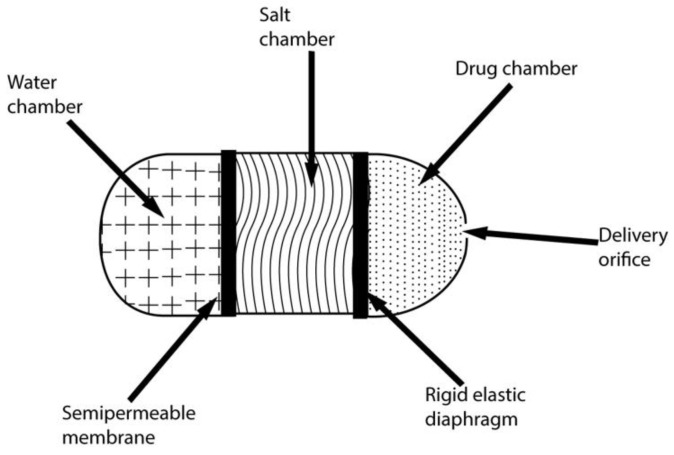
The Rose–Nelson pump.

The first implantable osmotic technique pump (shown in Figure 2) was reported in 1955 by Rose and Nelson, Australian physiologists. The pump was designed to deliver drugs into the guts of animals (sheep and cattle) [65]. A semi-permeable wall and water chamber surround an elastic diaphragm consisting of a section of excess salt. These two chambers are separated by a semi-permeable barrier. This creates a difference in gradient and osmotic pressure; thus, the water tends to move towards the chamber of salt from its chamber. As water enters the salt chamber, the volume of the salt chamber increases; hence, the diaphragm starts to distend, resulting in the pumping of the drug. The drug is then pumped out of the device [66]. A comparison of different systems is presented in Table 2.

#### 4.1.2. The Higuchi–Leeper Pump

**Figure 3:**
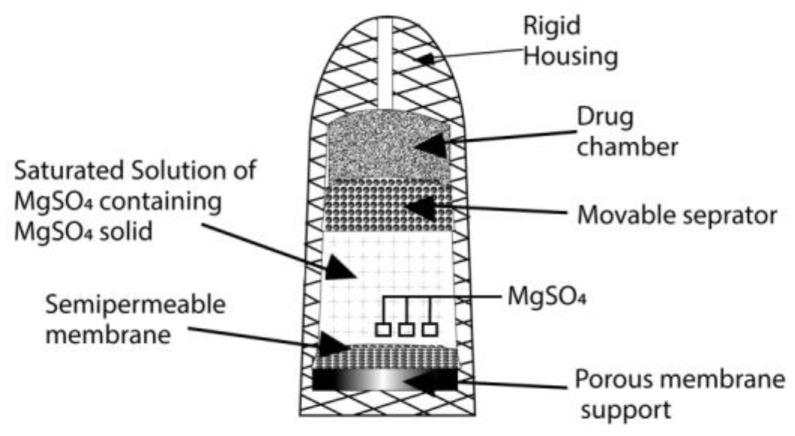
The Higuchi–Leeper pump.

In the 1970s, Alza Corporation introduced the Higuchi–Leeper pump (Figure 3), a simplified form of the Rose–Nelson pump [67]. The water chamber is absent in the Higuchi–Leeper pump. Instead, the water required for device activation is drawn from the environment in the device’s surroundings [68]. This is a beneficial modification, as it enables the storage of prepared and drug-loaded pumps for a month [69].

#### 4.1.3. The Higuchi–Theeuwes Pump

**Figure 4:**
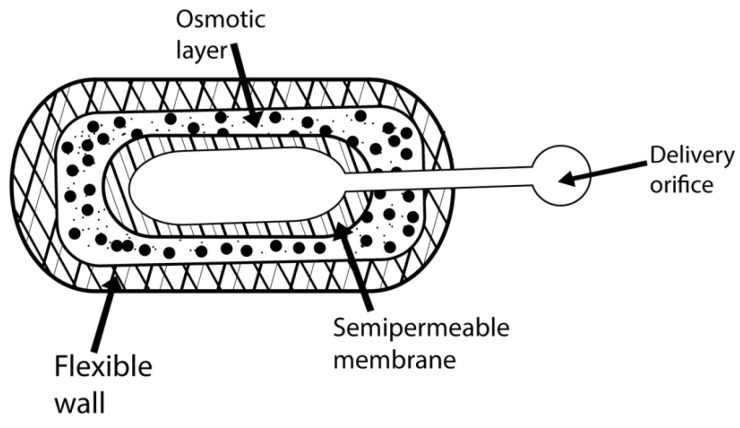
The Higuchi–Theeuwes pump.

In the 1970s, Higuchi and Theeuwes introduced a pump that had an outer casing made up of a rigid semi-permeable membrane (Figure 4). This pump is similar in form to the Rose–Nelson pump. The drug is loaded into the device before use [70]. The rate of drug release from the device when it is operated depends on the outer membrane’s permeation ability, and a time course that the salt has set is followed [71].

#### 4.1.4. The Implantable Mini Osmotic Pump

The mini pump consists of a reservoir that forms the drug layer, an osmotic agent layer, and a semi-permeable wall [72].

#### 4.1.5. The Alzet Osmotic Pump

For research purposes in laboratory animals, implantable Alzet mini pumps are utilized [73]. A thermoplastic elastomer made of hydrocarbon forms a collapsible reservoir, and around it is a coating of the osmotic agent. Over this layer, another coating is present: a semi-permeable membrane made of a cellulose ester blend. The drug is loaded into the reservoir. Water enters through a semi-permeable membrane and reaches the osmotic agent layer, which creates pressure on the inside and thus releases the drug out of the device. The drug release rate depends on the water volume permeating through the selectively permeable wall and the drug concentration in the solution. This rate can be controlled by controlling the permeation of the semi-permeable walls. Volumes of 100 μL, 200 μL, and 2 mL are the capacities in which the device is available, and it provides rates of drug delivery in the range 0.11–10 μL/h [13,73].

### 4.2. Oral Systems

#### 4.2.1. Single-Chamber Osmotic Systems

##### Elementary Osmotic Pump (EOP)

**Figure 5:**
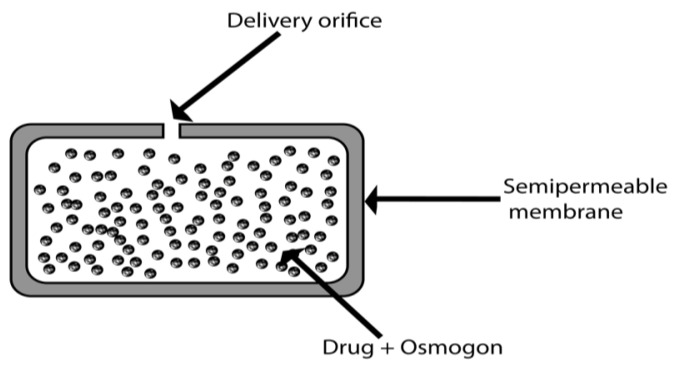
The structure of an EOP.

An EOP is a new system for drug delivery by an osmotic system invented by Theuwes in 1974. The release rate can be controlled by controlling the permeation ability of the semi-permeable membrane and the formulation characteristics [74]. In the formation of this device, the drug is compressed into a tablet, and then a coating of a semi-permeable cellulose acetate membrane is formed around it [75]. An orifice of about 0.5 to 1.5 mm is drilled in this membrane (Figure 5). A mechanical drill can be used for this purpose, but it can also be carried out by laser drilling using a CO_2_ laser beam with a 10.6-micron wavelength. When the device is put into operation, water enters through a semi-permeable wall by imbibition into the core, creating osmotic pressure. The drug solution volume is proportional to the solvent volume [76]. The rate of drug release is zero-order, which means that it is constant. The development of zero-order rates from the system requires a half-hour to one-hour lag phase before continuous delivery begins. About 60–80% of the drug has a constant drug release rate. Drugs with moderate water solubility are considered suitable for this system [77].

#### 4.2.2. Multichambered Osmotic Pumps

##### Push–Pull Osmotic Pump

**Figure 6:**
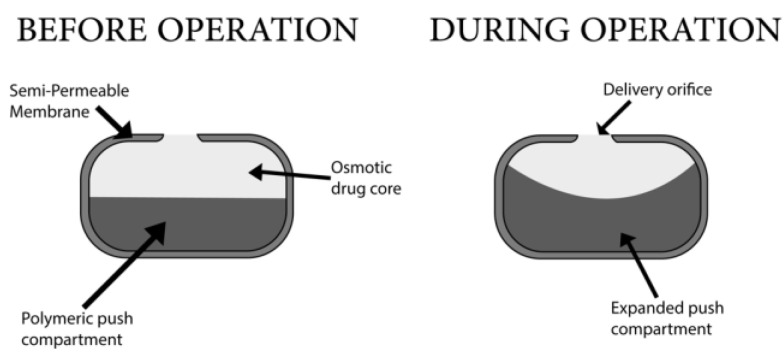
Push–pull osmotic pump.

This system has two compartments and an elastic diaphragm separating these compartments. Alza Corporation introduced this system [78]. The drug is contained in the upper compartment and is linked to the outer environment through an orifice for drug delivery. The lower compartment does not have an orifice, and it contains an osmotic agent. The drug compartment holds 60–80% of this tablet type, and the osmotic compartment holds 20–40% of the whole tablet weight [79]. Upon contact with aqueous surroundings, water imbibition occurs in both compartments, and the lower compartment containing no orifice starts to expand (Figure 6). This results in the diaphragm pushing toward the upper compartment, and the drug is thus released through the orifice. The lower compartment draws water into the device, causing expansion and forcing drugs through the orifice [80]. The push layer may be composed of carbopol (20–40%). The release of drugs in the local area is a disadvantage, and the cost of this device is high [25]. Some of the market-available pumps were summarized in Table 3.

##### Sandwiched Osmotic Tablet

**Figure 7:**
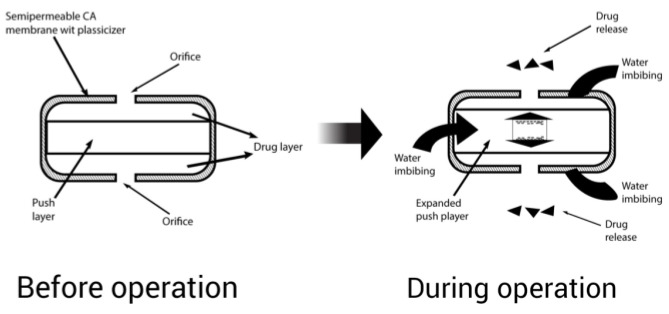
Sandwiched osmotic tablet.

This system consists of two compartments of drugs and one sandwiched compartment of polymers that acts as a push layer. The two drug compartments have orifices for drug delivery [81]. When this system comes into contact with an aqueous medium, the swelling of the middle-sandwiched layer starts to occur due to the presence of swelling agents, resulting in the delivery of the drug through two orifices present in both compartments (Figure 7). Drugs that cause local irritation of the gastric mucosa are suitable for this system [82].

##### Osmotic Pump with a Non-Expanding Second Chamber

This system has a second non-expanding chamber [83]. Based on the second chamber’s function, two subgroups can be created. This device has an area into which the second chamber dilutes the solution of the drug that will be released [84]. This helpful technique overcomes the risk of gastrointestinal irritation when a saturated solution of the drug is released [31]. It consists of two chambers that are rigid in nature. An inert osmotic agent such as common salt or sugar is present in the first chamber, and the drug is loaded into the second chamber. When activated, water enters into both chambers via a selectively permeable membrane. The first chamber is connected via a hole to the second chamber, so the osmotic solution formed in the first chamber enters through this hole to the second chamber and dissolves with the drug solution. It is then released via a membrane that has a microporous structure [84].

#### 4.2.3. Specific Types

##### Controlled-Porosity Osmotic Pump

**Figure 8:**
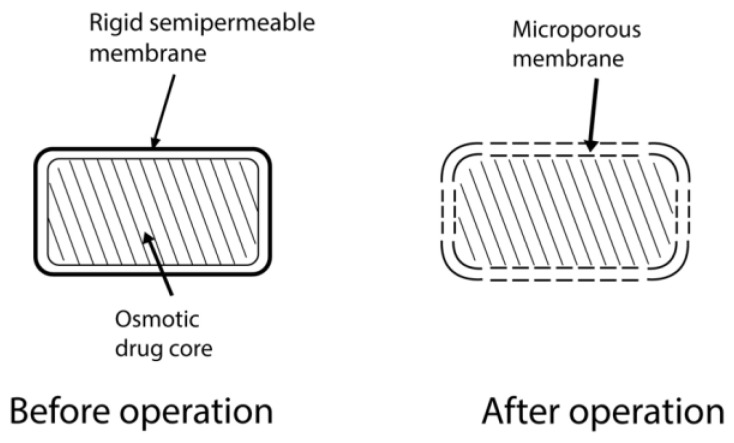
Controlled-porosity osmotic pump.

In 1985, Zenter presented the simplest osmotic system for the oral delivery of a drug. This osmotic pump tablet consists of a core compartment loaded with drugs with some water-soluble additives coated by an asymmetric insoluble membrane (Figure 8). The membrane has selective permeability for water only [85]. When exposed to the aqueous environment, water-soluble additives start dissolving and leaching out of the system, resulting in the formation of micropores in the membrane, giving it a spongelike appearance [45]. The microporous wall formed is then permeable to water and the drug in dissolved form. Sorbitol, urea, and sodium chloride are some of the pore-formers used [86]. Materials that can produce 5–95% pores and pore sizes in the range of 10–100 μm are beneficial. Different studies have been conducted to study the mechanism of drug release with moderate to high solubility, and some modulators of solubility are also being studied [87]. The thickness of the soluble portion and drug solubility, osmotic pressure, and coating surface area are the factors that affect the drug release rate from this system. However, this system does not depend on pH or physiological conditions. The designer of the device can control all of these factors [88].

##### Monolithic Osmotic System

This system includes agents that are soluble in water and are dispersed in a matrix of polymers [89]. Polymers form the capsule around the drug. Upon contact with aqueous surroundings, the active ingredients draw water into the system, resulting in the rupture of the polymeric capsule around the drug, thus releasing the drug out of the system [90]. These phenomena move serially from the outer material side towards the inner side of the polymer matrix. The release is driven by osmotic pressure at a zero-order rate [91,92].

##### Colon-Targeted Oral Osmotic System [93]

This is used to target the colon for drug delivery [94]. It may contain a hard gelatin capsule containing 5 or 6 osmotic units of the push–push system, or it may have a single agent for osmotic pressure. The dissolution of the gelatin capsule starts when this system is exposed to a gastric environment [95]. To prevent stomach fluids from entering the system, an enteric coating is present [96]. This coating dissolves in the intestines, and fluid thus enters the system and reaches the push compartment, causing it to swell [97]. A flowable gel is pushed out through the orifice in the drug compartment. The water transport rate controls the release rate of the gel. Drugs that are poorly soluble in water can be delivered via an osmotic system by forming a complex of the drug with cyclodextrin [98,99].

##### Osmotically Bursting Osmotic Pump

Baker invented this system of controlling drug release. The orifice for drug delivery may either be absent in this system or present at a very small size [100]. When water enters the system in the aqueous environment, hydraulic pressure forms. This ruptures the system, releasing its contents into the surroundings. Pulsatile release can be achieved when using this system. By controlling the thickness and area of the semi-permeable membrane, the drug release rate can be controlled in this system [101].

##### Asymmetrical-Membrane Osmotic Tablet

An asymmetric membrane enclosing a core loaded with the drug in the form of a capsule comprises this system’s structure [102]. The capsule wall is composed of polymers that are insoluble in water [103]. These capsules do not show immediate dissolution and cause prolonged drug release [104].

##### Liquid Oral Osmotic System

Liquid forms of drugs are delivered via this design, providing a high rate and extent of absorption and giving the advantages of extended release. A liquid drug formulation that is self-emulsifying and lipophilic is considered desirable for this system [105]. A semi-permeable membrane encloses an osmotic push layer. A drug layer represents the structure of this design, which is available in three forms (hard cap, soft cap, and delayed liquid bolus) [106]. Water imbibition activates the osmotic agent layer on exposure to an aqueous environment. This expands the push layer, and hydrostatic pressure pushes the drug out of the device’s system. Alza designs these systems. This system increases the permeation ability of drugs [107].

##### Effervescent Osmotic Tablet

Carbon dioxide is produced upon the reaction of an effervescent compound with acid, and such a combination is used in this system [108]. A drug in precipitate form is delivered due to the expansion caused by gas, and orifices are also not blocked. This benefits drugs for which solubility is poor at a low pH. As the pH in the gastric area is low, such drugs may precipitate, resulting in orifice blockage. A commonly used material is sodium bicarbonate [109].

##### Multiparticulate Delayed-Release System

In this type of design, pure drugs in pellets that may or may not contain osmotic agents are enclosed in a selectively permeable membrane [110]. When exposed to water, a saturated drug solution is formed when the water reaches the core. The membrane expands rapidly, resulting in pore formation. The drug is released at a zero-order rate. The rates of dissolution and lag time for this system are being studied [111]. Bioavailability can also be increased by using this technique [112].

##### Self-Emulsified Osmotic Tablet

Self-emulsified agents are added to the system when dealing with insoluble drugs or drugs with slight solubility [113]. Around 40% of drugs are poorly soluble in aqueous media. Drug bioavailability is enhanced by using self-emulsifying agents, resulting in a stable plasma concentration. For example, surfactants such as glycerol (sorbitan oleate, stearate, or laurate) and oxyethylene glyceryl ricinoleate are used [114].

##### Telescopic Capsule for Delayed Release [77]

**Figure 9:**
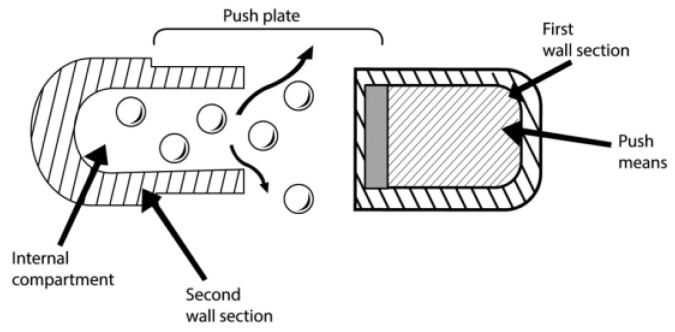
Telescopic capsule for delayed release.

This system consists of a waxy layer separating two chambers. The drug is contained in the first chamber with an orifice, while an osmotic agent is present in the second chamber. An automated mechanism fills the drug. The capsule cap contains a bilayer tablet, while the cap’s closed end includes a barrier (Figure 9). The capsule’s open end is fitted with a filled vessel. Compression is applied to this assembly [115,116]. When water enters the system, the expansion of the osmotic layer occurs, resulting in pressure generation on the wall of sections. The drug volume is constant in the delay period to minimize the fluid’s net flow into the core of the system [53].

**Table 3:** Marketed osmosis-based products [117,118,119].

Product Name	Active Pharmaceutical Ingredients	Osmotic System Type
Oral Products		
UT-15C	Treprostinil Diethanolamine	EOP
LCP-Lerc	Lercanidipine	EOP
Ditropan XL	Oxybutynin Chloride	EOP
Altoprev	Lovastatin	EOP
Flexeril XL	Cyclobenzaprine	EOP
Elafax XR	Venlafaxine HCl	EOP
Tegretol XL	Carbamazepine	EOP
Osmosin	Indomethacin	EOP
Teosona Sol	Theophylline	EOP
Allegra D 24 h	Pseudoephedrine HCl Fexofenadine HCl	EOP
Loremex	Pseudoephedrine HCl Loratadin	EOP
Mildugen D	Pseudoephedrine HCl Astemizol	EOP
Efidac 24bromphenirmine	Pseudoephedrine HCl Brompheniramin	EOP
Volmax	Albuterol	EOP
Acutrim	Phenylpropanolamine	EOP
Osmoran	Ranitidine HCl	EOP
Teczem	Enalapril Diltiazem	Controlled Porosity Osmotic Pump
Tiamate	Diltiazem HCl	Controlled Porosity Osmotic Pump
ActoPlus XR	Metformin HCl Pioglitazone HCl	Controlled Porosity Osmotic Pump
Acu System C	Vitamine C	Controlled Porosity Osmotic Pump
Covera HS	Verapamil HCl	Push–pull Osmotic Pump
DynaCirc CR	Isradipine	Push–pull Osmotic Pump
Minipress XL	Prazosin	Push–pull Osmotic Pump
Procardia XL	Nifedipine	Push–pull Osmotic Pump
Glucotrol XL	Glipizide	Push–pull Osmotic Pump
Cardura CR	Doxazosin Mesylate	Push–pull Osmotic Pump
Oxycontin	Oxycodone	Push–pull Osmotic Pump
Jusnista	Hydromorphone	Push–pull Osmotic Pump
Invega	Paliperidone	Push–pull Osmotic Pump
Topamax	Topiramate	Push-Stick Osmotic Pump
Concerta	Methylphenidate HCl	Push-Stick Osmotic Pump
Implantable Products		
ChronogesicTM	Sufentanil	Implantable osmotic system
Viadur	Leuprolide Acetate	Implantable osmotic system

## 5. Conclusions

Modified versions of conventional dosage forms have been introduced to overcome the limitations of traditional dosage forms. The revised versions are known as controlled-release drug delivery systems. Among the various controlled-release systems, osmotic pumps are widely accepted and employed to control drug delivery release. As is discussed in this review, osmotic pressure is the principle of this system. The drug release pattern, which is independent of pH and physiological factors, is one of the significant benefits of this system, making predetermined drug release rates possible.

## Figures and Tables

**Table 1:** Advantages and disadvantages of osmotic delivery systems.

Advantages	Disadvantages
After the administration of the system as intended, first comes a phase known as the lag phase, which occurs in the initial phase, and then a zero-order rate of drug release is achieved [30].	There might be a risk of dose dumping, resulting in a greater concentration of the drug in the blood than required with potential for toxicity [31].
Side effects are reduced when using this technique.	With implantable pumps, there is a chance of rapidly developing tolerance.
By using this technique, a modified form as a delayed or pulsed-type system for introducing drugs into the body can be formulated when required [32].	If the patient’s immune system is hypersensitive, it may cause a hypersensitivity reaction.
In an osmotic system, the release of drugs is driven by osmotic pressure, so it is not dependent on physiological factors of the gastric region, such as pH, and is also independent of hydrodynamic factors [20].	The integrity of this system and its required consistency are difficult to achieve [33].
The release rate is independent of the drug.	There might be a risk of film defects when there is a problem or poor performance in the coating process. The critical step is the size of the orifice through which the drug is delivered, and this has a direct impact on the drug delivery system’s performance [6,14].
The correlation between in vitro dissolution and in vivo bioavailability is high when this system is employed [34].	If any toxicity or poisoning occurs, it is impossible to withdraw or to retrieve it from the body system [35].
This system is easily formulated and is also simple to handle.	
When implanted or given orally, these systems provide the prolonged or sustained release of drugs, thereby reducing the frequency of drug doses to be taken by patient and improving patient compliance [36,37].	
These systems provide sustained drug release and achieve a steady-state and uniform drug concentration in the blood to provide a therapeutic effect for a prolonged period [35].	

**Table 2:** Comparison of different osmotic delivery systems [7,67,68,69].

Osmotic System	Similarity	Differences/Drawbacks
EOP	All the osmotic drug delivery systems have a semi-permeable membrane that controls the flow of water and has an osmotic core.	It consists of only one orifice and the drug is released only in solution form. Suitable only for water-soluble drugs. Drug release follows only zero-order kinetics.
Rose-Nelson Pump		One of the major drawbacks of this pump is that whenever the pump comes into contact with water, its osmotic action begins due to the presence of a semi-permeable membrane. For this reason, the pump is stored empty and in air-tight containers and must be loaded with water before use.
Push–pull		A push–pull system is better than an EOP system as this system can release drugs both in suspension and solution form. Thus, this system is preferred for highly soluble drugs such as oxybutynin hydrochloride and for very poorly soluble drugs such as glipizide and nifedipine. Meanwhile, the system has a drawback in that a complicated drilling technique is involved in drilling the orifice in the pump.
Push-stick		A push-stick is a longitudinally compressed bilayer tablet system. It consists of a single large orifice and osmotic agents in the push layer. Drug is released from the system as it comes in contact with water and gets moistened, followed by disintegration and then dissolution.
Controlled Porosity and Single Composition Osmotic Tablet (SCOT)		This system consists of a single-layer tablet with no drilled holes; hence, the drug is released from the system through cracks in the form of a wet mass or solution. Compared to other systems, this system significantly reduces gastric irritation. The drug is released from the whole surface of the device (not only from a single orifice). Furthermore, no complex laser drilling, such as that required in a push–pull system, is required since the orifices are formed in situ.
L-OROS		These are soft-gel capsules consisting of only one orifice, and the drug is released in liquid form.

## Data Availability

Not applicable.

## References

[1] Keraliya R.A., Patel C., Patel P., Keraliya V., Soni T.G., Patel R.C., Patel M. (2012). Osmotic drug delivery system as a part of modified release dosage form. Int. Sch. Res. Not..

[2] Kaparissides, Costas, Alexandridou S., Kotti K., Chaitidou S. (2006). Recent advances in novel drug delivery systems. J. Nanotechnol. Online.

[3] Patel J., Parikh S., Patel S. (2021). Comprehensive review on osmotic drug delivery system. World J. Pharm. Res..

[4] Bruschi M.L. (2015). Strategies to Modify the Drug Release from Pharmaceutical Systems.

[5] Cheng L., Gao S., Ouyang D., Wang H., Wang Y., Pan W., Yang X. (2018). Release Mechanism Between Ion Osmotic Pressure and Drug Release in Ionic-Driven Osmotic Pump Tablets (I). AAPS PharmSciTech.

[6] Siddique W., Zaman M., Sarfraz R.M., Butt M.H., Rehman A.U., Fassih N., Albadrani G.M., Bayram R., Alfaifi M.Y., Abdel-Daim M.M. (2022). The Development of Eletriptan Hydrobromide Immediate Release Buccal Films Using Central Composite Rotatable Design: An In Vivo and In Vitro Approach. Polymers.

[7] Arafat M. (2015). Approaches to achieve an oral controlled release drug delivery system using polymers: A recent review. Int. J. Pharm. Pharm. Sci..

[8] Ratnaparkhi M., Gupta Jyoti P.J.T. (2013). Sustained release oral drug delivery system—An overview. Int. J. Pharm. Res..

[9] Farooqi S., Yousuf R.I., Shoaib M.H., Ahmed K., Ansar S., Husain T. (2020). Quality by Design (QbD)-Based Numerical and Graphical Optimization Technique for the Development of Osmotic Pump Controlled-Release Metoclopramide HCl Tablets. Drug Des. Dev. Ther..

[10] Swathi B., Manichandrika K.P., Niharika R., Pravalika G., Sahithya D., Meghana M. (2019). Formulation and evaluation of quinidine osmotic drug delivery system. Int. J. Adv. Res. Med. Pharm. Sci..

[11] Hanif M., Zaman M., Chaurasiya V. (2015). Polymers used in buccal film: A review. Des. Monomers Polym..

[12] Flavia L., Keckeis V. (2020). Advances in drug delivery systems: Work in progress still needed?. Int. J. Pharm..

[13] Shirole P.U., Patil P.B., Bachhav R.S. (2020). Review on Osmotic Drug Delivery System. IJRAR-Int. J. Res. Anal. Rev. (IJRAR).

[14] Patel H., Parikh V.P. (2017). An overview of osmotic drug delivery system: An update review. Int. J. Bioassays.

[15] Sahoo C.K., Rao S.R., Sudhakar M. (2015). Evaluation of controlled porosity osmotic pump tablets: A Review. Res. J. Pharm. Technol..

[16] Sharma A., Kumar D., Painuly N. (2018). A review on osmotically controlled drug delivery systems. Asian J. Pharm. Res. Dev..

[17] Gundu R., Pekamwar S., Shelke S., Kulkarni D., Gadade D., Shep S. (2022). Development and pharmacokinetic evaluation of osmotically controlled drug delivery system of Valganciclovir HCl for potential application in the treatment of CMV retinitis. Drug Deliv. Transl. Res..

[18] Zaman M., Siddique W., Waheed S., Sarfraz R., Mahmood A., Qureshi J., Iqbal J., Chughtai F., Rahman M.U., Khalid U. (2015). Hydrogels, their applications and polymers used for hydrogels: A review. Int. J. Biol. Pharm. Allied Sci..

[19] Srikonda S., Kotamraj P., Barclay B. (2006). Osmotic controlled drug delivery systems. Design of Controlled Release Drug Delivery Systems.

[20] Santus G., Baker R.W. (1995). Osmotic drug delivery: A review of the patent literature. J. Control. Release.

[21] Sareen R., Jain N., Kumar D.J. (2012). An insight to osmotic drug delivery. Curr. Drug Deliv..

[22] Harper D.J., Milo C.F. (2002). Osmotic Pump Drug Delivery Systems and Methods. U.S. Patent.

[23] Patel K.N., Mehta T.A. (2013). A review on oral osmotically driven systems. Int. J. Pharm. Pharm. Sci..

[24] Prajapati H.M., Prajapati S.T., Patel C.N. (2012). A review on recent innovation in osmotically controlled drug delivery system. Int. J. Pharm. Res. Bio-Sci..

[25] Cortese R., Theeuwes F. (1982). Osmotic Device with Hydrogel Driving Member. U.S. Patent.

[26] Malaterre V., Ogorka J., Loggia N., Gurny R.J.I. (2009). Approach to design push–pull osmotic pumps. Int. J. Pharm..

[27] Gundu R., Pekamwar S., Shelke S., Kulkarni D., Shep S. (2021). Development, optimization and pharmacokinetic evaluation of biphasic extended-release osmotic drug delivery system of trospium chloride for promising application in treatment of overactive bladder. Future J. Pharm. Sci..

[28] Kumaravelrajan R., Narayanan N., Suba V.J.L. (2011). Development and evaluation of controlled porosity osmotic pump for Nifedipine and Metoprolol combination. Lipids Health Dis..

[29] Wong P.S., Theeuwes F., Barclay B.L., Dealey M.H. (1995). Osmotic Dosage System for Liquid Drug Delivery. U.S. Patent.

[30] Rastogi S., Vaya N., Mishra B.J.E. (1995). Osmotic pump: A novel concept in rate controlled oral drug delivery. East. Pharm..

[31] Verma R., Mishra B., Garg S. (2000). Osmotically controlled oral drug delivery. Drug Dev. Ind. Pharm..

[32] Farheen F., Bharadwaj S. (2014). A review on osmotically regulated systems. PharmaTutor.

[33] Udawant S.J.W. (2015). A review: Osmotic drug delivery system. World J. Pharm. Res..

[34] Malaterre V., Ogorka J., Loggia N., Gurny R.J. (2009). Oral osmotically driven systems: 30 years of development and clinical use. Eur. J. Pharm. Biopharm..

[35] Babu A., Ratna P.R.V. (2010). Controlled-porosity osmotic pump tablets—An overview. Asian J. Pharm. Res. Health Care.

[36] Verma R.K., Garg S.J. (2004). Development and evaluation of osmotically controlled oral drug delivery system of glipizide. Eur. J. Pharm. Biopharm..

[37] Rudnic E.M., Burnside B.A., Flanner H.H., Wassink S.E., Couch R.A., Pinkett J.E. (2000). Osmotic Drug Delivery System. U.S. Patent.

[38] Singla D., Hari Kumar S., Nirmala G.J. (2012). Osmotic pump drug delivery: A novel approach. Int. J. Res. Pharm. Chem..

[39] Okimoto K., Rajewski R.A., Stella V.J. (1999). Release of testosterone from an osmotic pump tablet utilizing (SBE) 7m-β-cyclodextrin as both a solubilizing and an osmotic pump agent. J. Control. Release.

[40] Herrlich S., Spieth S., Messner S., Zengerle R. (2012). Osmotic micropumps for drug delivery. Adv. Drug Deliv. Rev..

[41] Jerzewski R.L., Chien Y.W. (2017). Osmotic drug delivery. Treatise on Controlled Drug Delivery.

[42] Banerjee A., Verma P.R.P., Gore S. (2015). Controlled porosity solubility modulated osmotic pump tablets of Gliclazide. AAPS PharmSciTech.

[43] Siew A. (2013). Controlling drug release through osmotic systems. Pharm. Technol..

[44] Lindstedt B., Ragnarsson G., Hjärtstam J. (1989). Osmotic pumping as a release mechanism for membrane-coated drug formulations. J. Control. Release.

[45] Makhija S.N., Vavia P.R. (2003). Controlled porosity osmotic pump-based controlled release systems of pseudoephedrine: I. Cellulose acetate as a semipermeable membrane. J. Control. Release.

[46] Seminoff L.A., Zentner G.M. (1989). Cellulosic Coating. European Patent.

[47] Ahmed K., Shoaib M.H., Yousuf R.I., Qazi F., Anwer S., Nasiri M.I., Mahmood Z.A. (2018). Use of Opadry^®^ CA—A cellulose acetate/polyethylene glycol system for rate-controlled osmotic drug delivery of highly soluble antispastic agent Eperisone HCl. Adv. Polym. Technol..

[48] Sanap S.L., Savkare A.D. (2014). Controlled porosity osmotic pump: A review. Int. J. Pharm. Res. Dev..

[49] Markovsky E., Baabur-Cohen H., Eldar-Boock A., Omer L., Tiram G., Ferber S., Ofek P., Polyak D., Scomparin A., Satchi-Fainaro R. (2012). Administration, distribution, metabolism and elimination of polymer therapeutics. J. Control. Release.

[50] Ghosh T., Ghosh A.J. (2011). Drug delivery through osmotic systems—An overview. J. Appl. Pharm. Sci..

[51] Mathur M., Mishra R. (2016). A review on osmotic pump drug delivery system. Pharma Sci. Monit..

[52] Zentner G.M., Rork G.S., Himmelstein K. (1985). The controlled porosity osmotic pump. J. Control. Release.

[53] Thulasiramaraju T., Reddy S.R., Patnaik N.A., Kumar K.S. (2013). Osmotic drug delivery system: A promising drug delivery Technology. AJRC.

[54] Verma R.K., Krishna D.M., Garg S.J. (2002). Formulation aspects in the development of osmotically controlled oral drug delivery systems. J. Control. Release.

[55] Singh K., Walia M.K., Agarwal G., Harikumar S.J. (2013). Osmotic pump drug delivery system: A noval approach. J. Drug Deliv. Ther..

[56] Asghar F., Ahmad S., Gul M., Malik T. (2020). Mucoadhesive system to enhance drug activity containing Flurbiprofen as model drug. Mucoadhesive Syst. Enhanc. Drug Act. Contain. Flurbiprofen Model Drug.

[57] Patel P.M., Yadav J. (2018). Osmotic Controlled Drug Delivery System: A Review. Pharma Sci. Monit..

[58] Venkateswarlu B.S., Pasupathi C., Pasupathi A., Jaykar B., Chandira R.M., Palanisamy P. (2019). Osmotic drug delivery system: A review. J. Drug Deliv. Ther..

[59] Sharma D., Kumar D., Singh M., Singh G., Rathore M.S. (2012). A review on novel osmotically controlled drug delivery system. Indian J. Pharm..

[60] Verma R., Mishra B.J.P. (1999). Studies on formulation and evaluation of oral osmotic pumps of nimesulide. Pharmazie.

[61] Theeuwes F., Saunders R.J., Mefford W.S. (1978). Process for Forming Outlet Passageways In pills Using a Laser. U.S. Patents.

[62] Li N., Fan L., Wu B., Dai G., Jiang C., Guo Y., Wang D. (2019). Preparation and in vitro/in vivo evaluation of azilsartan osmotic pump tablets based on the preformulation investigation. Drug Dev. Ind. Pharm..

[63] Chen C.-M., Lee D.-Y., Xie J. (1998). Controlled Release Formulation for Water Insoluble Drugs in Which a Passageway is Formed In Situ. U.S. Patents.

[64] Gawai N.M., Aher S.S., B R. (2016). Saudagar. A Review on Drug Category Suitable for Monolithic Osmotic Tablet. Res. J. Pharm. Dos. Technol..

[65] Rose S., Nelson J. (1955). A continuous long-term injector. Aust. J. Exp. Biol. Med. Sci..

[66] Gohel M., Parikh R., Shah N. (2009). Osmotic Drug Delivery: An Update. World J. Pharm. Res..

[67] Higuchi T., Leeper H. (1976). Osmotic Dispenser. U.S. Patent.

[68] Higuchi T. (1973). Osmotic Dispenser with Collapsible Supply Container. U.S. Patent.

[69] Umamaheswari A. (2012). Formulation and Evaluation of Controlled Porosity Osmotic Tablets of Lornoxicam. Ph.D. Thesis.

[70] Theeuwes F., Yum S. (1976). Principles of the design and operation of generic osmotic pumps for the delivery of semisolid or liquid drug formulations. Ann. Biomed. Eng..

[71] Theeuwes F. (1978). Osmotic System for Delivering Selected Beneficial agents Having Varying Degrees of Solubility. U.S. Patnet.

[72] Capozza R., Eckenhoff B., Yum S. (1977). Design and performance of the implantable osmotic minipump. J. Med. Eng. Technol..

[73] Kerenyi S.Z., Hartgraves S.L. (1987). Premature excess release from the Alzet osmotic pump. Pharmacol. Biochem. Behav..

[74] Ouyang D., Nie S., Li W., Guo H., Liu H., Pan W. (2005). Design and evaluation of compound metformin/glipizide elementary osmotic pump tablets. J. Pharm. Pharmacol..

[75] Lieberman H.A., Lachman L., Schwartz J.B., Dekker M. (1980). Pharmaceutical Dosage Forms: Tablets.

[76] Theeuwes F. (1975). Elementary osmotic pump. J. Pharm. Sci..

[77] Murkute R.S., Wanare R.S., Dongre K.J. (2012). A Review: Recent Scenario on Osmotic Controlled Drug Delivery System. Res. J. Pharm. Technol..

[78] Prabakaran D., Singh P., Kanaujia P., Jaganathan K., Rawat A., Vyas S.P. (2004). Modified push–pull osmotic system for simultaneous delivery of theophylline and salbutamol: Development and in vitro characterization. Int. J. Pharm..

[79] Wong P.S., Barclay B., Deters J.C., Theeuwes F. (1986). Osmotic Device with Dual Thermodynamic Activity. US Patent.

[80] Wakode R., Bhanushali R., Bajaj A. (2008). Development and evaluation of push–pull based osmotic delivery system for pramipexole. PDA J. Pharm. Sci. Technol..

[81] Kumaravelrajan R., Narayanan N., Suba V., Bhaskar K. (2010). Simultaneous delivery of Nifedipine and Metoprolol tartarate using sandwiched osmotic pump tablet system. Int. J. Pharm..

[82] Mohanty S., Sahu M., Sirisha A. (2014). Osmotic pump: A novel approach to control drug delivery. Indo Am. J. Pharm. Res..

[83] Pujara N., Thacker A.P., Dudhat K.R., Patel N.V., Parmar R.B. (2012). Osmotically Controlled Oral Drug Delivery Systems: A Novel Approach. Inventi Rapid NDDS.

[84] Parashar B., Maurya B., Yadav V., Sharma L. (2012). A review on osmotically regulated devices. Pharma Innov..

[85] Syed S.M. (2015). Osmotic Drug Delivery System: An Overview. Drug Deliv. Syst..

[86] Gupta N.R., Mishal A., Bhosle Y., Shetty S. (2014). A review on recent innovation in osmotically controlled drug delivery system. Indian J. Pharm. Biol. Res..

[87] Haslam J.L., Rork G.S. (1989). Controlled Porosity Osmotic Pump. U.S. Patent.

[88] Sahoo C.K., Sahoo N.K., Rao S.R.M., Sudhakar M., Satyanarayana K. (2015). A review on controlled porosity osmotic pump tablets and its evaluation. Bull. Fac. Pharm. Cairo Univ..

[89] Oakley J.A., Shaw K.J., Docker P.T., Dyer C.E., Greenman J., Greenway G.M., Haswell S.J. (2009). Development of a bi-functional silica monolith for electro-osmotic pumping and DNA clean-up/extraction using gel-supported reagents in a microfluidic device. Lab Chip.

[90] Liu L., Khang G., Rhee J.M., Lee H.B. (2000). Monolithic osmotic tablet system for nifedipine delivery. J. Control. Release.

[91] Liu L., Che B. (2006). Preparation of monolithic osmotic pump system by coating the indented core tablet. Eur. J. Pharm. Biopharm..

[92] Patra C.N., Swain S., Sruti J., Patro A.P., Panigrahi K.C., Beg S., Rao M.E. (2013). Osmotic drug delivery systems: Basics and design approaches. Pat. Drug Deliv. Formul..

[93] Shah N., Patel N., Patel K., Patel D. (2012). A review on osmotically controlled oral drug delivery systems. J. Pharm. Sci. Bio Res..

[94] Liu H., Yang X.-G., Nie S.-F., Wei L.-L., Zhou L.-L., Liu H., Tang R., Pan W.-S. (2007). Chitosan-based controlled porosity osmotic pump for colon-specific delivery system: Screening of formulation variables and in vitro investigation. Int. J. Pharm..

[95] Nie X., Wang B., Hu R., Lu W., Chen J., Liu S., Jin D., Sun C., Gao S., Guo Y.J.A.P. (2020). Development and Evaluation of Controlled and Simultaneous Release of Compound Danshen Based on a Novel Colon-Specific Osmotic Pump Capsule. AAPS PharmsciTech.

[96] Li M., Shen Q., Lu W., Chen J., Yu L., Liu S., Nie X., Shao L., Liu Y., Gao S. (2020). Development and evaluation of controlled release of metformin hydrochloride for improving the oral bioavailability based on a novel enteric osmotic pump capsule. J. Drug Deliv. Sci. Technol..

[97] Amidon S., Brown J.E., Dave V.S. (2015). Colon-targeted oral drug delivery systems: Design trends and approaches. AAPS Pharmscitech.

[98] Mene H.R., Mene N.R., Parakh D.R., Ingale T.B., Magar D.R., Mangale M.R. (2016). Formulation aspects in development of controlled porosity osmotic pump tablet. Pharm. Biol. Eval..

[99] Siddique W., Zaman M., Khan R., Butt M.H., Muhammad K., Salawi A., Safhi A.Y., Sabei F.Y. (2022). Formulation and Evaluation of Hydroxypropyl Methylcellulose Based Topical Gel Loaded with Ganciclovir. Lat. Am. J. Pharm..

[100] Gupta B.P., Thakur N., Jain N.P., Banweer J., Jain S. (2010). Osmotically controlled drug delivery system with associated drugs. JPPS.

[101] Piyush G., Pankaj R., Dabeer A., Ayaj A. (2010). A review on osmotically regulated devices. Int. J. Pharm. Life Sci..

[102] Thombre A., DeNoto A., Gibbes D. (1999). Delivery of glipizide from asymmetric membrane capsules using encapsulated excipients. J. Control. Release.

[103] Suman S., Chauhan M. (2011). Asymmetric Membrane Capsule: New Prospects in Osmotic Delivery. Int. J. Drug Deliv..

[104] Kolli P., Kancharla S., Gopaiah K.V. (2021). Osmotic release tablets formulation and evaluationof ace inhibitor molecule. Int. J. Pharm. Drug Anal..

[105] Kushner J., Lamba M., Stock T., Wang R., Nemeth M.A., Alvey C., Chen R., DeMatteo V., Blanchard A. (2020). Development and validation of a Level A in-vitro in-vivo correlation for tofacitinib modified-release tablets using extrudable core system osmotic delivery technology. Eur. J. Pharm. Sci..

[106] Dong L., Shafi K., Wan J., Wong P. A novel osmotic delivery system: L-OROS Soft cap. Proceedings of the International Symposium on Controlled Release of Bioactive Materials.

[107] Zentner G.M., Rork G.S., Himmelstein K.J. (1990). Controlled porosity Osmotic Pump. U.S. Patent.

[108] Li X.-D., Nie S.-F., Wu L. (2004). Studies on controlled release effervescent osmotic pump tablets from Traditional Chinese Medicine Compound Recipe. J. Control. Release.

[109] Raja R.K., Vasu N.V., Anka R.A. (2015). Osmotic controlled drug delivery system: A review. Int. J. Pharm. Sci..

[110] Haslam J.L., Rork G.S. (1989). Multiparticulate Controlled Porosity Osmotic Pump. U.S. Patent.

[111] Schultz P., Kleinebudde P. (1997). A new multiparticulate delayed release system.: Part I: Dissolution properties and release mechanism. J. Control. Release.

[112] Roy P., Shahiwala A. (2009). Multiparticulate formulation approach to pulsatile drug delivery: Current perspectives. J. Control. Release.

[113] Kommana N., Bharti K., Surekha D.B., Thokala S., Mishra B. (2020). Development, optimization and evaluation of losartan potassium loaded Self Emulsifying Drug Delivery System. J. Drug Deliv. Sci. Technol..

[114] Wei L., Li J., Guo L., Nie S., Pan W., Sun P., Liu H. (2007). Investigations of a novel self-emulsifying osmotic pump tablet containing carvedilol. Drug Dev. Ind. Pharm..

[115] Khandagale P.M., Bhairav B., Saudagar R. (2017). Osmotically Controlled Drug Delivery System-A Novel Approach. Asian J. Res. Pharm. Sci..

[116] Xue Y., Yu S., Wang H., Liang J., Peng J., Li J., Yang X., Pan W. (2015). Design of a timed and controlled release osmotic pump system of atenolol. Drug Dev. Ind. Pharm..

[117] Patil P., Uphade K., Saudagar R. (2018). A Review: Osmotic Drug Delivery System. Pharma Sci. Monit..

[118] Qiu Y., Lee P. (2017). Rational design of oral modified-release drug delivery systems. Developing Solid Oral Dosage Forms.

[119] Khatri N., Nikam S., Bilandi A. (2016). Oral osmotic drug delivery system: A review. Int. J. Pharm. Sci. Res..

